# Metagenomic and metatranscriptomic inventories of the lower Amazon River, May 2011

**DOI:** 10.1186/s40168-015-0099-0

**Published:** 2015-09-10

**Authors:** Brandon M. Satinsky, Caroline S. Fortunato, Mary Doherty, Christa B. Smith, Shalabh Sharma, Nicholas D. Ward, Alex V. Krusche, Patricia L. Yager, Jeffrey E. Richey, Mary Ann Moran, Byron C. Crump

**Affiliations:** Department of Microbiology, University of Georgia, Athens, GA 30602 USA; Horn Point, Laboratory University of Maryland Center for Environmental Science, Cambridge, MD 21612 USA; Department of Marine Sciences, University of Georgia, Athens, GA 30605-3636 USA; School of Oceanography, University of Washington, Seattle, WA 98112 USA; CENA-USP, Avenida Centenário 303, 13416-000 Piracicaba, São Paulo Brazil; College of Earth, Ocean, and Atmospheric Science, Oregon State University, CEOAS Admin Bldg, Corvallis, OR 97331-5503 USA

**Keywords:** Amazon River, Metagenomics, Metatranscriptomics, Internal standards, Microbial communities

## Abstract

**Background:**

The Amazon River runs nearly 6500 km across the South American continent before emptying into the western tropical North Atlantic Ocean. In terms of both volume and watershed area, it is the world’s largest riverine system, affecting elemental cycling on a global scale.

**Results:**

A quantitative inventory of genes and transcripts benchmarked with internal standards was obtained at five stations in the lower Amazon River during May 2011. At each station, metagenomes and metatranscriptomes were obtained in duplicate for two microbial size fractions (free-living, 0.2 to 2.0 μm; particle-associated, 2.0 to 297 μm) using 150 × 150 paired-end Illumina sequencing. Forty eight sample datasets were obtained, averaging 15 × 10^6^ potential protein-encoding reads each (730 × 10^6^ total). Prokaryotic metagenomes and metatranscriptomes were dominated by members of the phyla *Actinobacteria*, *Planctomycetes*, *Betaproteobacteria*, *Verrucomicrobia*, *Nitrospirae*, and *Acidobacteria*. The actinobacterium SCGC AAA027-L06 reference genome recruited the greatest number of reads overall, with this single bin contributing an average of 50 billion genes and 500 million transcripts per liter of river water. Several dominant taxa were unevenly distributed between the free-living and particle-associated size fractions, such as a particle-associated bias for reads binning to planctomycete *Schlesneria paludicola* and a free-living bias for actinobacterium SCGC AAA027-L06. Gene expression ratios (transcripts to gene copy ratio) increased downstream from Óbidos to Macapá and Belém, indicating higher per cell activity of Amazon River bacteria and archaea as river water approached the ocean.

**Conclusion:**

This inventory of riverine microbial genes and transcripts, benchmarked with internal standards for full quantitation, provides an unparalleled window into microbial taxa and functions in the globally important Amazon River ecosystem.

**Electronic supplementary material:**

The online version of this article (doi:10.1186/s40168-015-0099-0) contains supplementary material, which is available to authorized users.

## Background

The Amazon River is the world’s largest riverine system, formed by a network of tributaries draining Andean and lowland basins [1]. Understanding the fate of materials transported through the Amazon River will help to better quantify its impact on global elemental cycles. As is the case for other large tropical rivers, the Amazon drains extensive floodplains and other continental areas of high primary and secondary production [2] and contributes significantly to organic matter and nutrient export to the ocean [3, 4]. Processes occurring within the river also drive large fluxes of methane and carbon dioxide to the atmosphere [5–7].

**Fig. 1:**
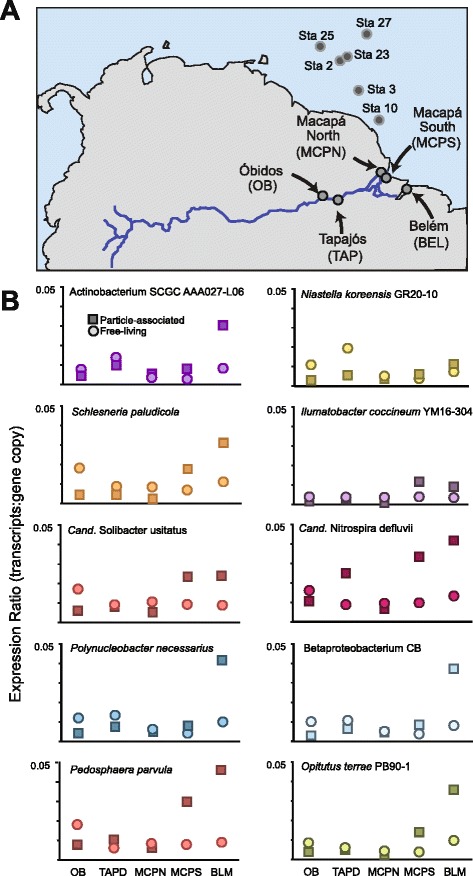
**a** Location of sampling sites in the Amazon River in May 2011. Sampling locations for previous metagenomic and metatranscriptomic datasets in the offshore plume of the Amazon (June, 2010) are also indicated. **b** Expression ratios (transcript to gene copy ratio) for the ten reference genomes recruiting the most reads from the metagenomes. Ratios are shown by station and size fraction (*squares*, particle-associated; *circles*, free-living)

To obtain insights into the microbial processes of the lower Amazon River, we inventoried the genes and transcripts at five stations in May 2011 (Fig. 1). This study complements a previous analysis of the genes and transcripts of the Amazon Plume conducted in 2010, in which microbial expression ratios were determined at six stations across the mid- and outer plume. A previous metagenomic study in the Amazon main stem at a site several hundred kilometers upriver from our starting point at Óbidos revealed an actinobacteria-dominated ecosystem with a high abundance of genes for heterotrophic carbon processing [8].

Here, metagenomic and metatranscriptomic sequences were obtained by Illumina sequencing, using 150 × 150 bp overlapping paired-end reads. Whereas community genomic data have typically been analyzed within a relative framework (i.e., percent of metagenome and percent of metatranscriptome), the approach used here incorporated known copy numbers of internal standards added at the initiation of sample processing [9]. This allowed transcripts and genes to be inventoried within an absolute framework (transcripts L^−1^, gene copies L^−1^, and transcript to gene copy ratios), facilitating comparisons of gene expression levels and regulatory responses among taxa and between river locations.

Measurements were made at five stations along the lower Amazon River including the historic downstream gauging station, Óbidos, the clear water Tapajós tributary, and the three primary channels near the Amazon River mouth. For each station, metagenomes and metatranscriptomes were obtained in duplicate for two discrete size fractions (free-living, 0.2 to 2.0 μm; particle-associated, 2.0 to 297 μm), resulting in 40 datasets (5 stations × 2 nucleic acid types × 2 size fractions × 2 replicates). At the Tapajós station, an additional set of filters were collected from the surface water for comparison with the sample collected at 50 % of the river depth. Following quality control (removal of poor quality reads, removal of ribosomal RNA (rRNAs) from metatranscriptomes, removal of internal standards, and joining of overlapping 150 bp paired ends), 760 million potential protein-encoding reads were obtained and analyzed for taxonomy and function.

## Methods

Detailed sample collection and processing methodology can be found in Additional file 1. The five sampling sites were located at Óbidos, the Tapajós River confluence, the north and south Macapá channels, and Belém (Fig. 1a, Additional file 2). Water samples were collected at 50 % of water column depth at each station, which ranged from 10–33 m among the stations, and microbial cells were collected by filtration and preserved in RNAlater (Applied Biosystems, Austin, TX). During sample processing, internal standards consisting of two different ~1000 base RNA standards [10, 11] and *T. thermophilus* HB8 genomic DNA standard [9] were added to each sample prior to cell lysis. The samples collected for metatranscriptomics were processed by extracting total RNA from the collected filters following the removal of RNAlater, treating the extracted total RNA with DNase to remove residual DNA, depleting rRNA through subtractive hybridization with community specific biotinylated nucleotide probes, linearly amplifying the remaining transcripts, and making double-stranded complementary DNA (cDNA) for library preparation and sequencing. The metagenomic samples were processed by extracting DNA and removing residual proteins and RNA. Following sample processing, cDNA or DNA was sheared and libraries were constructed for paired-end sequencing (150 × 150) using the Illumina HiSeq 2500 platform.

From a total of 48 samples, we obtained 1.27 × 10^9^ raw sequences. Following quality control, 0.94 × 10^9^ reads with a mean length of 212 bp were obtained. Internal standards were quantified and removed, along with any remaining rRNA sequences, leaving 0.73 × 10^9^ possible protein-encoding reads. These were annotated against the RefSeq protein database using RAPSearch2 [12], and abundance per liter was calculated based on internal standard recovery following methods in Satinsky et al. [9] (Additional file 2).

Biological and chemical data measured concurrently with sample collection included temperature, depth, conductivity, bacterial abundance, dissolved inorganic carbon concentrations, and chlorophyll concentrations (Additional file 2). Datasets describing the organic chemistry and bacterial respiration were collected at each of the lower stations [13–15].

## Quality assurance

The PANDAseq program [16] was used to join the paired-end Illumina reads using all default parameters except for the threshold score, which was set at a value stricter than the default (0.8). The FASTX-Toolkit [http://hannonlab.cshl.edu/fastx_toolkit/index.html] was used for quality control of the paired reads. Ribosomal RNA and internal standard sequences were identified in the metatranscriptomes using a Blastn search against a custom database containing representative rRNA sequences and internal standard sequences; sequences with a bit score ≥50 were identified as either rRNA or internal standards and removed from the datasets. Internal standards were identified in metagenomes by first performing a Blastn search (bit score cutoff ≥ 50) against the *T. thermophilus* HB8 genome. Hits were subsequently queried against the RefSeq protein database using Blastx (bit score cutoff ≥ 40) to identify and quantify *T. thermophilus* HB8 protein-encoding reads, and these reads were removed from the datasets prior to data analysis and deposition.

## Initial findings

Metagenomic and metatranscriptomic reads from the five Amazon River stations were assigned to bacterial, archaeal, eukaryotic, and viral taxa based on the best hits to reference genomes in the RefSeq protein database. Bacterial reads dominated the sequence collections, accounting for >90 % of quality-controlled reads in the metagenomes and >60 % in the metatranscriptomes (Table 1). Eukaryotic reads amounted to only 3 % of the genes but 9 % of the free-living and 30 % of the particle-associated transcript pools. Archaea accounted for 3 % of the genes and about 10 % of transcripts. Viral-like sequences (presumably representing ongoing viral infections; few free viral particles would be captured on the filters used for sample collection) accounted for 3 % of the community genes and 1 % of the transcripts (Table 1).

**Table 1:** Library types and reads obtained in the Amazon River May 2011 microbial gene and transcript inventories, as part of the Amazon Continuum Project

Data type	Metagenomes (community DNA)		Metatranscriptomes (community mRNA)		
Stations sampled	6		6		
Size fractions sampled	2		2		
Replicates	2		2		
Samples	24		24		
Raw reads	8.85 × 10^8^		1.73 × 10^9^		
Joined reads post QC	3.54 × 10^8^		6.17 × 10^8^		
Average joined read length (bp)	218		222		
rRNA reads	–		1.52 × 10^8^		
Potential protein-encoding reads	3.52 × 10^8^		3.78 × 10^8^		
Average abundance (genes L^−1^ or transcripts L^−1^)	Free-living	Particle-associated		Free-living	Particle-associated
Bacterial	3.70 × 10^12^	6.18 × 10^12^		3.24 × 10^10^	6.17 × 10^10^
Archaeal	1.25 × 10^11^	1.94 × 10^11^		4.70 × 10^9^	8.07 × 10^9^
Eukaryotic	9.56 × 10^10^	1.88 × 10^11^		3.44 × 10^9^	3.04 × 10^10^
Viral	1.39 × 10^11^	1.31 × 10^11^		4.50 × 10^8^	5.42 × 10^8^

**Fig. 2:**
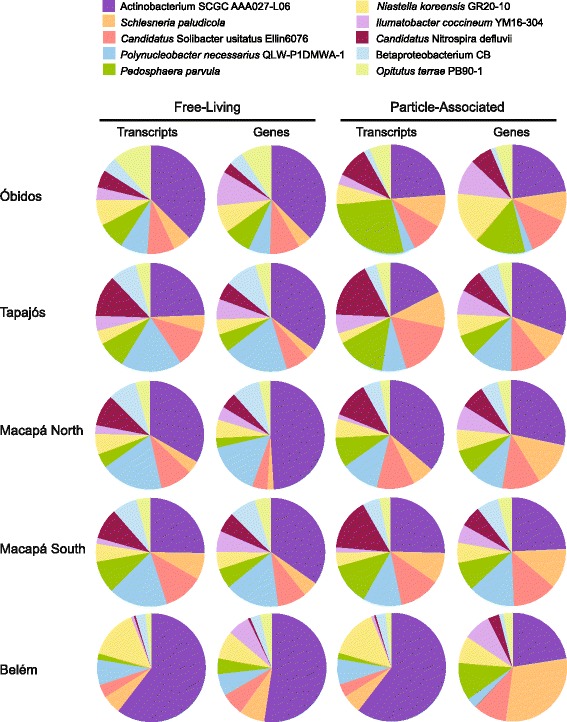
Relative abundance of genes and transcripts for the ten reference genomes recruiting the most metagenomic reads

The ten reference genomes recruiting the highest number of reads were in the phyla *Actinobacteria* (actinobacterium SCGC AAA027-L06, *Ilumatobacter coccineum* YM16-304), *Planctomycetes* (*Schlesneria paludicola*), *Betaproteobacteria* (*Polynucleobacter necessarius* subsp. *asymbioticus* QLW-P1DMWA-1, betaproteobacterium CB), *Verrucomicrobia* (*Pedosphaera parvula*, *Opitutus terrae* PB90-1), *Nitrospirae* (*Candidatus* Nitrospira defluvii), and *Acidobacteria* (*Candidatus* Solibacter usitatus Ellin6076) (Fig. 2). Reads binning to the actinobacterium SCGC AAA027-L06 genome were common in every sample and were particularly abundant at the upriver stations. At Tapajós, for example, SCGC AAA027-L06 contributed ~7 × 10^11^ genes L^−1^ and ~1 × 10^10^ transcripts L^−1^, or 7 and 11 % of the prokaryotic metagenome and metatranscriptome at that site. The *Actinobacteria* phylum is frequently the most numerically dominant in freshwater environments, and the acI-B1 tribe that includes SCGC AAA027-L06 [17] is one of the more abundant clades [18]. Multiple thaumarchaeote and methylotroph genomes were also among the higher-recruiting bins.

We estimated average genome equivalents per liter of river water for the ten most dominant taxa based on genes L^−1^ in the reference genome bin (calculated from recoveries of the internal genomic standard) and the number of protein-encoding genes in each genome (Table 2). Genomes of actinobacterium SCGC AAA027-L06 and betaproteobacterium *P. necessarius* subsp. *asymbioticus* QLW-P1DMWA-1 were the most abundant, with 2.0 × 10^9^ and 3.5 × 10^8^ genomes, respectively, in an average liter of Amazon River water. Most of the dominant taxa contributed more genomes to the particle-associated community than the free-living community. The most extreme examples were planctomycete *S. paludicola*, which was fourfold more abundant in the larger size class, and nitrite-oxidizing *Candidatus* Nitrospira defluvii, which was threefold more abundant. This pattern is consistent with knowledge of the biology of these groups, as *Planctomycetes* have been observed on marine aggregates and other surfaces [19, 20] and are considered specialists in degrading complex organic matter, whereas members of the genus *Nitrospira* form cell clusters during active growth [21]. Looking across all dominant taxa, there was a positive correlation between genome size and extent of association with particulate material, a pattern consistent with previous studies of marine planktonic bacteria [22, 23].

**Table 2:** Gene and transcript inventories for the ten reference genome bins recruiting the highest number of metagenome reads

Reference genome	Phylum	Mean transcripts L^−1^	Mean genes L^−1^	Mean % of transcripts	Mean % of genes	Genome size^a^	PA genome equivalents L^−1^	FL genome equivalents L^−1^	Total genome equivalents L^−1^
Actinobacterium SCGC AAA027-L06	*Actinobacteria*	4.84 × 10^9^	4.97 × 10^11^	11.2	7.1	1244	1.92 × 10^8^	2.08 × 10^8^	4.00 × 10^8^
*Schlesneria paludicola*	*Planctomycetes*	1.30 × 10^9^	1.51 × 10^11^	1.7	2.5	7033	1.71 × 10^7^	4.41 × 10^6^	2.15 × 10^7^
*Polynucleobacter necessarius asym*.	*Betaproteobacteria*	1.55 × 10^9^	1.49 × 10^11^	1.1	1.3	2077	4.19 × 10^7^	2.73 × 10^7^	6.91 × 10^7^
*Candidatus* Solibacter usitatus Ellin6076	*Acidobacteria*	1.16 × 10^9^	1.44 × 10^11^	1.4	0.9	7998	1.31 × 10^7^	5.53 × 10^6^	1.86 × 10^7^
*Pedosphaera parvula*	*Verrucomicrobia*	2.00 × 10^9^	1.16 × 10^11^	1.0	1.2	6510	1.25 × 10^7^	5.36 × 10^6^	1.79 × 10^7^
*Niastella koreensis* GR20-10	*Bacteriodetes*	1.04 × 10^9^	1.12 × 10^11^	2.7	1.4	7136	9.54 × 10^6^	6.09 × 10^6^	1.56 × 10^7^
*Ilumatobacter coccineum* YM16-304	*Actinobacteria*	4.44 × 10^8^	1.10 × 10^11^	0.2	1.2	4233	1.56 × 10^7^	1.04 × 10^7^	2.60 × 10^7^
*Candidatus* Nitrospira defluvii	*Nitrospirae*	1.44 × 10^9^	7.42 × 10^10^	0.7	0.3	3938	1.45 × 10^7^	4.34 × 10^6^	1.88 × 10^7^
Betaproteobacterium CB	*Betaproteobacteria*	5.38 × 10^8^	7.27 × 10^10^	0.5	0.4	2034	2.11 × 10^7^	1.46 × 10^7^	3.57 × 10^7^
*Opitutus terrae* PB90-1	*Verrucomicrobia*	6.94 × 10^8^	6.75 × 10^10^	0.4	0.5	4612	7.61 × 10^6^	7.04 × 10^6^	1.46 × 10^7^
Sum		1.50 × 10^10^	1.49 × 10^12^	20.8	16.8				6.38 × 10^8b^

Data were averaged across five locations (Fig. 1a) sampled in the lower Amazon River in May 2011

^a^Number of protein-encoding genes in the reference genome

^b^The top ten taxa averaged 16.8 % of the genes in Amazon River water. Applying this same proportion to the number of genome equivalents contributed by the top ten taxa (6.38 × 10^8^), the total genome equivalent is estimated at 3.80 × 10^9^ genomes L^−1^. By comparison, direct epifluorescence microscopy counts indicate 3.46 × 10^9^ cells L^−1^

We were able to assess the accuracy of gene count calculations based on the internal genomic standard by comparing genome equivalent estimates to direct cell count data obtained by epifluorescence microscopy. The average number of genome equivalents in Amazon River water was estimated to be 3.80 × 10^9^ genomes L^−1^, calculated by extrapolating from the sum of the genome equivalents of the top ten taxa (6.38 × 10^8^; Table 2) and assuming they account for 16.8 % of the total genome equivalents (the same as their percent contribution to total genes; Table 2). In excellent agreement with this internal standard-based calculation, direct cell counts indicated an average of 3.46 × 10^9^ cells L^−1^ (Additional file 2).

Calculations of the expression ratio (defined as the ratio of transcripts to gene copy) allowed comparisons of transcriptional activity among taxa, and by river location and size fraction. Of the ten most dominant prokaryotic reference genomes, all but nitrite-oxidizing *Candidatus* Nitrospira defluvii showed higher average transcripts to gene copy ratios in the larger size class, indicative of more active cells when associated with aggregates or particulate material. Within this overall pattern, however, expression ratios were consistently higher for free-living cells in the upriver stations at Óbidos and Tapajós and at the Macapá-South station. At Macapá-North and Belém, as well as in offshore Amazon Plume stations sampled in a previous study [11], particle-associated prokaryotic cells were considerably more transcriptionally active (Fig. 1b). An additional collection was made of surface water at the Tapajós station to compare with the 50 % water depth sample, in order to assess depth-related differences in river microbial communities that could indicate water column substructure. The composition of the surface and 50 % depth metagenomes and metatranscriptomes were highly similar (Additional file 2: Figure S1).

## Future directions

The Amazon basin plays a central role in global nutrient cycling, and the rainforest surrounding the river is responsible for nearly 10 % of global primary production [24]. At its mouth, the Amazon discharges water at a rate greater than the next six largest global rivers combined. To better understand the diversity and metabolic activity of microbial communities within this extremely large river system and its oceanic plume, four high-throughput metagenomic and metatranscriptomic sequence datasets are being produced as part of the ANACONDAS and ROCA projects (http://amazoncontinuum.org). The June 2010 plume dataset has been published [11]. Two additional datasets consisting of concurrently sampled plume and river collections in July 2012 are in progress. These high-coverage, size-discrete, and replicated datasets are all benchmarked with internal genomic and messenger RNA (mRNA) standards. Future analysis will focus on expression of biogeochemically relevant genes mediating key transformations in the carbon, nitrogen, and phosphorus cycles and the physiological and environmental factors regulating expression levels [10, 25].

## Availability of supporting data

Sequences from this May 2011 Amazon Continuum study are available from NCBI under accession numbers SRP039390 (metagenomes) and SRP037995 (metatranscriptomes). The NCBI sequences are fastq files from which internal standard sequences (metagenomes and metatranscriptomes) and rRNA sequences (metatranscriptomes only) have been removed prior to deposition. Metadata accompanying the omics datasets are provided in Additional file 2. ANACONDAS and ROCA project data are also available at the BCO-DMO data repository (http://www.bco-dmo.org/project/2097).

## Additional files

Additional file 1:
**Detailed methods.** Description of metagenome and metatranscriptome sample processing, sequencing, and data analysis, including internal standard additions and analyses. **Figure S1**. Hierarchical clustering of the Bray-Curtis dissimilarities in the taxonomic binning of transcripts from two microbial size fractions from 50 % water depth at each of five locations in the Amazon River and from the surface water at the Tapajós station. Additional file 2:
**Metadata.** Metadata accompanying the metagenomic and metatranscriptomic datasets, including station locations, environmental conditions, and library sizes and statistics.

## Abbreviations

bp: base pairs
cDNA: complementary DNA
mRNA: messenger RNA
rRNA: ribosomal RNA
