# Supplementary material for: Metagenomic and metatranscriptomic inventories of the lower Amazon River, May 2011
Source: Microbiome. 2015 Sep 10;3:39. doi: 10.1186/s40168-015-0099-0 (PMC4564970; doi:10.1186/s40168-015-0099-0)
Supplement: Additional file 1: — Detailed methods. Description of metagenome and metatranscriptome sample processing, sequencing, and data analysis, including internal standard additions and analyses. Figure S1. Hierarchical clustering of the Bray-Curtis dissimilarities in the taxonomic binning of transcripts from two microbial size fractions from 50 % water depth at each of five locations in the Amazon River and from the surface water at the Tapajós station. [file 40168_2015_99_MOESM1_ESM.pdf]

Metagenomic and metatranscriptomic inventories of the lower Amazon River, May 2011

Brandon M. Satinsky, Caroline Fortunato, Mary Doherty, Christa B. Smith, Shalabh Sharma, Nicholas D. Ward, Alex V. Krusche, Patricia L. Yager, Jeffrey E. Richey, Mary Ann Moran, and Byron C. Crump

Detailed Methods

Sample Collection

At each of the five stations selected for analysis, water was collected at 50% of the river depth by pumping water with a Shurflo submersible pump through a 297 μm stainless steel screen and collecting the water in 20 L carboys on the deck of the boat. In addition, at the Tapajos site water was also collected from the surface via the same methodology. The collected water was sequentially filtered using a Masterflex peristaltic pump through a 2.0 μm pore-size, 142 mm diameter polycarbonate (PCTE) membrane filter (Sterlitech Corporation, Kent, CWA)and a 0.22 μm pore-size, 142 mm diameter Supor membrane filter (Pall, Port Washington, NY). Duplicate samples were collected for each membrane size/type for total community metagenomics and metatranscriptomics. After filtration, membranes were immediately submerged in RNAlater (Applied Biosystems, Austin, TX) in sterile 15 ml conical tubes, refrigerated until returning to shore, and then stored in a freezer until extraction. All filtration and stabilization was completed within 30 min of water collection, and the volume of filtrate passed through each membrane was recorded. Additionally, samples were collected and preserved for flow cytometry. After staining with 1X SYBR Green II and incubating in the dark for at least 10 minutes, samples were analyzed using a Beckman Coulter Cyan flow cytometer with an excitation wavelength of 488 nm and emission detection filter of 530 nm. Bacterial cells were quantified using a combination of light scatter and fluorescence detection with a bead standard of known concentration used to calculate the sample volume counted by the instrument.

RNA Processing for Total Community Metatranscriptomes

Prior to RNA extraction, the filters were thawed, removed from the preservative solution, placed in Whirl-Pak bags (Nasco, Fort Artkinson, WI), and flash-frozen in liquid nitrogen. Frozen filters were broken into small pieces with a rubber mallet and transferred to a 50 mL lysis tube containing 10 mL of Denaturation Solution (Ambion), 500 μL of Plant RNA Isolation Aid (Ambion), 2 mL of sterilized zirconium beads (OPS Diagnostics), and internal standards[1]. Tubes were vortexed for 10 min to lyse cells, after which time the tubes were centrifuged for 1 min at 5,000 rpm. The lysates were transferred to a sterile 15 mL conical tube and then centrifuged for 5 min at 5,000 rpm. The clarified lysates were transferred to sterile 50 mL conical tubes and 3.5 mL of saturated phenol (pH 4.3) was added to each lysate and vortexed thoroughly. The tubes were centrifuged for 8 min at 12,000 x g, after which the non-viscous phase in each tube was transferred to a fresh 50 mL conical tube, and 3.5 mL of a phenol:chloroform solution (1:1, pH 5) was added and tube contents were mixed well. Following another 8 min centrifugation at 12,000 x g, the aqueous phase in each tube was transferred to a sterile 50 mL conical tube and 5 mL of chloroform:isoamyl alcohol solution was added. Tubes were vortexed and contents were centrifuged for 5 min at 12,000 x g. The final aqueous phase in each tube was transferred to a fresh 50 mL conical tube prior to the addition of an equal volume of 100% ethanol. Each mixture was homogenized by passage through a syringe several times. RNA purification was completed for each sample using the Direct-Zol RNA Kit (Zymo Research) according to manufacturer's protocol. Residual DNA was removed from the samples by two successive treatments with the Turbo DNA-free kit (Invitrogen, Carlsbad, CA). Ribosomal

RNA (rRNA) was selectively removed using community-specific biotinylated-rRNA probes prepared from DNA collected simultaneously [2]. To maximize the removal of rRNA, probes were created for Bacterial and Archaeal 16S and 23S rRNA and Eukaryotic 18S and 28S rRNA. Probe-bound rRNA was removed via hybridization to streptavidin-coated magnetic beads (New England Biolabs, Ipswich, MA), and successful removal of rRNA from the samples was confirmed using either an Experion automated electrophoresis system (Bio-Rad Laboratories, Hercules, CA) or a Bioanalyzer (Agilent Technologies, Santa Clara, CA). rRNA-depleted samples were linearly amplified using the MessageAmp II-Bacteria Kit (Applied Biosystems, Austin, TX), and amplified mRNA was converted into cDNA using the Superscript III First Strand synthesis system (Invitrogen, Carlsbad, CA) with random primers, followed by the NEBnext mRNA second strand synthesis module (New England Biolabs, Ipswich, MA), both according to manufacturer protocols. Synthesized cDNA was purified using the QIAquick PCR purification kit (Qiagen, Valencia, CA) followed by EtOH precipitation, resuspension in 100 µL of TE buffer, and storage at -80° C until library preparation for sequencing.

DNA Processing for Metagenomes

DNA was extracted and purified as previously described [3, 4] with some modification. Briefly, each filter was thawed, removed from RNAlater, and rinsed three times in autoclaved, filter-sterilized, 0.1% phosphate-buffered saline (PBS) to remove any residual RNAlater. Each filter was shattered as described above and placed in a tube containing DNA extraction buffer [DEB: 0.1 M Tris-HCl (pH 8), 0.1 M Na-EDTA (pH 8), 0.1 M $Na_2H_2PO_4$ (pH 8), 1.5 M NaCl, 5% CTAB]. All liquid from the rinses as well as the original RNAlater was pushed through a Sterivex-GP filter capsule (EMD Millipore, Billerica, MA), which was subsequently rinsed 3 times to salvage any lost cells. The capsule was opened and the filter sliced into pieces and added to the tube with the original membrane filter and an internal genomic DNA standard (described below). Following treatments with proteinase-K, lysozyme, and sodium dodecyl sulfate, DNA was purified via phenol:chloroform extraction and isopropanol precipitation.

Internal Standards

Omics processing included the addition of internal standards to allow for calculation of volume-based absolute copy numbers for each gene or transcript type, rather than just relative quantification (i.e., counts L^{-1} in addition to % of library) [1, 5]. Two mRNA standards that mimicked prokaryotic mRNAs were synthesized by in vitro transcription using a method modified previously described [1]. The standards were constructed by linearizing two custom synthesized vectors with a restriction enzyme. Each was purified by phenol:chloroform:isoamyl alcohol extraction and ethanol precipitation. Complete digestion of the vector was confirmed on a 1% agarose gel. The DNA fragment was then transcribed in vitro using the Riboprobe in vitro Transcription System (Promega, Madison, WI) according to the manufacturer’s protocol using a T7 RNA polymerase to create the two artificial transcripts that were each 1,006 nt in length. Residual DNA was removed using RQ1 RNase-Free DNase and the RNA was purified by phenol:chloroform:isoamyl alcohol extraction and ethanol precipitation. The RNA standards were quantified using the Quant-iT Ribogreen RNA Reagent and Kit (Invitrogen, Carlsbad, CA), and RNA nucleotide length was confirmed with a bioanalyzer. A known copy number of each standard was added independently to each lysis tube immediately prior to the addition of the sample filter.

The genomic internal standard consisted of *Thermus thermophilus* DSM7039 [HB8] genomic DNA (American Type Culture Collection, Manassas, VA) added immediately prior to cell lysis.

The amount of internal standards added was calculated based on estimated yield of DNA and total RNA as in [1].

Sequencing and Data Processing

cDNA and DNA was sheared ultrasonically to ~200-250 bp fragments and TruSeq libraries (Illumina Inc., San Diego, CA) were constructed for paired-end (150 x 150) sequencing using the Illumina HiSeq2500 platform (Illumina Inc., San Diego, CA). Following sequencing, reads were paired using PandaSeq (Masella *et al.,* 2012) and filtered with FastX toolkit (http://hannonlab.cshl.edu/fastx_toolkit/) with a minimum score cutoff of 20 over 80% of a read. From the paired, quality-controlled reads, internal standard sequences were quantified and removed, and any rRNA sequences were removed from the metatranscriptomes. Transcript and gene abundances as well as expression ratios were calculated as previously described [1].

References

1. Satinsky BM, Gifford SM, Crump BC, Moran MA: Use of Internal Standards for Quantitative Metatranscriptome and Metagenome Analysis. *Methods in Enzymolog*y 2013, 531:237-250.

2. Stewart FJ, Ottesen EA, DeLong EF: Development and quantitative analyses of a universal rRNA-subtraction protocol for microbial metatranscriptomics. *ISME J* 2010, 4:896–907.

3. Satinsky BM, Crump BC, Smith CB, Sharma S, Zielinski BL, Doherty M, Meng J, Sun S, Medeiros PM, Paul JH, Coles VJ, Yager PL, Moran MA: Microspatial gene expression patterns in the Amazon River Plume. *Proc Natl Acad Sci* 2014, 111:11085–90.

4. Satinsky BM, Zielinski BL, Doherty M, Smith CB, Sharma S, Paul JH, Crump BC, Moran MA: The Amazon continuum dataset: quantitative metagenomic and metatranscriptomic inventories of the Amazon River plume, June 2010. *Microbiome* 2014, 2:17.

5. Gifford SM, Sharma S, Rinta-Kanto JM, Moran MA: Quantitative analysis of a deeply sequenced marine microbial metatranscriptome. *ISME J* 2011, 5:461–472.

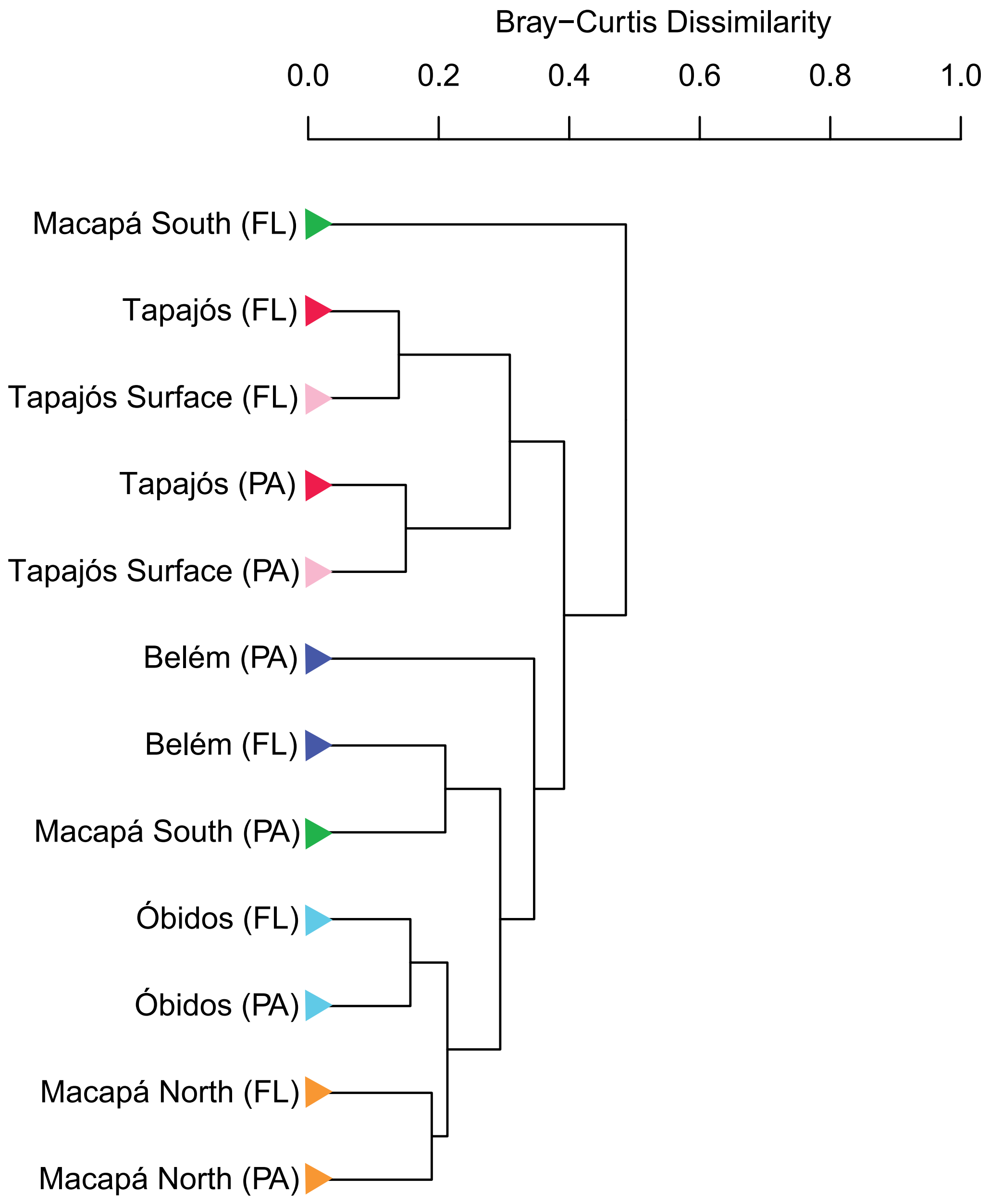

Figure S1.
Hierarchical clustering of the Bray-Curtis dissimilarities in the taxonomic binning of transcripts from two microbial size fractions from 50% water depth at each of five locations in the Amazon River and from the surface water at the Tapajós station (n=2 for all). Note the similarity in composition of the Tapajós surface and 50% water depth, indicative of a well-mixed water column.
